# Emotional experience of various types of cyberaggression by adolescents and youth

**DOI:** 10.1192/j.eurpsy.2021.254

**Published:** 2021-08-13

**Authors:** G. Soldatova, S. Chigarkova, A. Koshevaya

**Affiliations:** Faculty Of Psychology, Lomonosov Moscow State University, Moscow, Russian Federation

**Keywords:** emotional experience, adolescents, youth, cyberaggression

## Abstract

**Introduction:**

The integration of digital technologies into everyday life leads to transformation of various socio-cultural practices, including related to destructive behavior. Among them, cyberaggression holds a leading position, especially in younger generation, and causes direct lasting negative impact on the psychological state of participants and affects (Martínez-Monteagudo et al., 2019; Wright, Wachs, 2020).

**Objectives:**

The goal of the study is to analyze the relationship between offline and online aggressions and the strength of negative emotional experiences of adolescents and youth, as well as parents’ awareness of this experience with their children.

**Methods:**

The questionnaire was completed by 3395 people: 1554 adolescents aged 12-17 and 736 young people aged 18-30 from 8 federal districts of Russia.

**Results:**

Respondents of all generations (64-74%) believe that people are more likely to experience painful or hostile situations in real life than online. Nevertheless, every fourth respondent (19-23%) says that events on the Internet can cause as much anxiety as events in real life. The least emotionally significant situations are flaming and cyberhate. Trolling causes strong feelings in every third adolescent, cyberstalking in every fifth, cyberbullying in every second. Both trolling and cyberstalking make girls more upset than boys, this is true for adolescents (12-13 and 14-17 years old) and youth (χ2=19.01-67.21, p<0.01, V=0.16-0.30).

**Conclusions:**

Differences in emotional response to various types of cyberaggression require the development of differentiated approaches to the prevention of various situations of cyberaggression and the development of specific coping strategies in the collision with them. The reported study was funded by RFBR, project No. 20-013-00857.

**Disclosure:**

The reported study was funded by RFBR, project No. 20-013-00857.

